# Genome wide response to dietary tetradecylthioacetic acid supplementation in the heart of Atlantic Salmon (*Salmo salar* L)

**DOI:** 10.1186/1471-2164-13-180

**Published:** 2012-05-11

**Authors:** Fabian Grammes, Kjell-Arne Rørvik, Magny S Thomassen, Rolf K Berge, Harald Takle

**Affiliations:** 1 Institute of Animal and Aquaculture Sciences, Norwegian University of Life Sciences, P.O. Box 5003, N-1432 Ås-UMB, Norway; 2 NOFIMA, P.O. Box 5010, N-1432 Aas, Norway; 3 Institute of Medicine, Haukeland University Hospital, University of Bergen, N-5021 Bergen, Norway; 4 AVS Chile SA, Casilla 300, Puerto Varas, Chile

## Abstract

**Background:**

Under-dimensioned hearts causing functional problems are associated with higher mortality rates in intensive Atlantic salmon aquaculture. Previous studies have indicated that tetradecylthioacetic acid (TTA) induces cardiac growth and also stimulates transcription of peroxisome proliferator activated receptors (PPAR) *α*and *β*in the Atlantic salmon heart. Since cardiac and transcriptional responses to feed are of high interest in aquaculture, the objective of this study was to characterize the transcriptional mechanisms induced by TTA in the heart of Atlantic salmon.

**Results:**

Atlantic salmon were kept at sea for 17 weeks. During the first 8 weeks the fish received a TTA supplemented diet. Using microarrays, profound transcriptional effects were observed in the heart at the end of the experiment, 9 weeks after the feeding of TTA stopped. Approximately 90% of the significant genes were expressed higher in the TTA group. Hypergeometric testing revealed the over-representation of 35 gene ontology terms in the TTA fed group. The GO terms were generally categorized into cardiac performance, lipid catabolism, glycolysis and TCA cycle.

**Conclusions:**

Our results indicate that TTA has profound effects on cardiac performance based on results from microarray and qRT-PCR analysis. The gene expression profile favors a scenario of ”physiological”lright hypertrophy recognized by increased oxidative fatty acid metabolism, glycolysis and TCA cycle activity as well as cardiac growth and contractility in the heart ventricle. Increased cardiac efficiency may offer significant benefits in the demanding Aquaculture situations.

## Background

High levels of dietary lipids are used in commercial Atlantic salmon diets to promote rapid growth and as a inexpensive source of energy. These high lipid levels may promote excess lipid deposition in the viscera and the muscle, thereby reducing the market quality of the fish. Thus, tetradecylthioacetic acid (TTA: CH_3_-(CH_2_)_13_-S−CH_2_-COOH) has been tested for aquaculture nutrition, initially to increase lipid catabolism and thereby reducing lipid deposition [1]. However, beneficial effects on cardiac growth and disease resistance have also been addressed.

TTA is a modified fatty acid (FA) that possesses a sulfur atom in the *β*position. Like a normal FA, TTA can be converted to co-enzyme A thioester, but further catabolism by *β*-oxidation does not occur. This lack of metabolism is likely to determine the biological effects of TTA. Biological effects of TTA have been the focus of extensive research in rodents and also in humans. The most important findings from these experiments are that TTA increases the mitochondrial and peroxisomal *β*-oxidation and possesses hypolipidemic effects. In addition, TTA acts as an antioxidant *in vivo* and can modulate the inflammatory response (reviewed in [2]). Cell culture experiments demonstrated that TTA can act as a ligand for all Peroxisome proliferator activated receptors (PPARs) [3,4], which are ligand-activated transcription factors. Upon ligand activation PPARs heterodimerizes with retinoic-x-acid receptor (RXR) and have been shown to regulate the expression of genes involved in fatty acid metabolism, cell differentiation, development and inflammation (reviewed in [5]). Arguably most of the biological effects of TTA are mediated through activation of PPARs.

Studies addressing the biological effects of TTA in Atlantic salmon have demonstrated that TTA increases *β*-oxidation in the liver [1] and white muscle [6]. Furthermore, TTA reduces the secretion of triacylglycerides from Atlantic salmon hepatocytes *in vitro*[7] and has been shown to increase the expression of genes associated with fat metabolism in the liver and the heart ventricle [1,8]. Previous results also suggested that TTA stimulates the transcription of PPAR*α*and *β* in the heart [8,9], thus indicating that TTA affects the metabolism in Atlantic salmon through activation of PPARs, similar to the mechanism known from rodents. Interestingly, this activation of PPARs may have been related to increased survival after a natural outbreak of a heart related viral disease in Atlantic salmon [8,9]. In mammals, cardiac activation of PPARs has yielded substantial attention due to the fact that PPARs have been proven to be major regulators of cardiac metabolism [10-13]. In addition, PPAR agonists have been reported to exert beneficial effects by attenuating the pathogenesis of heart failure and atherosclerosis [14,15].

Poor development of the outer muscle layer, atherosclerosis and metabolic dysfunction have been related to under-dimensioned hearts and reduced cardiac function in Atlantic salmon aquaculture, consequently resulting in increased mortality [16]. Therefore, methods to improve cardiac metabolism and performance in fish are needed; something which has been sparsely studied. It appears that, similar to the mammalian heart, the oxidative cardiac metabolism in fish depends on the metabolism of fatty acids and glucose [17].

This study aims to characterize the cardiac transcriptional response of Atlantic salmon to a TTA supplemented diet. A feeding trial was conducted in sea, feeding a control and TTA supplemented diet during the first 8 weeks and only control diet for the subsequent 9 weeks of the experiment. Fish were sampled both at the end of the TTA feeding period (*8.weeks*) and at the end of the experiment (*17.weeks*). Our results show that administration of TTA to Atlantic salmon resulted in a marked change of cardiac gene expression. The expression profile suggests that TTA induces cardiac fatty acid oxidation, glycolysis, TCA cycle and contractility as well as cardiac growth.

## Results

### Production data

Atlantic salmon that were fed with 0.25% TTA had significantly lower fat content in the muscle at the *8.weeks* sampling point, and showed a tendency for increased mean relative heart weight (Table 1). No significant effect of TTA on fish weight was detected. During the experiment none of the dietary groups showed higher mortality than the control group. In the heart ventricles, 120.2 *μgTTA*/*gTissue*was detected at the *8.weeks* sampling point in the TTA group, while 1.2 *μgTTA*/*gTissue* was detected in the control group. Based on the TTA measurements of a group fed a higher (0.5% w/w) TTA diet from the same trial, we can assume that the TTA levels in the heart ventricles at the *17.weeks* sampling point were no different to the control group (see Additional file 1: Table S1).

**Table 1:** Effect of TTA on Atlantic salmon production parameters

	**Start sampling**	***8.weeks***		***17.weeks***	
		**Control**	**0.25% TTA**	**Control**	**0.25% TTA**
Weight[g]	102 ± 5	166 ±1	165 ±1	438 ±4	440 ±2
CF^1^	1.2 ± 0.1	1.1 ±0.1	1.1 ± 0.1	1.2 ± 0.1	1.2 ± 0.1
LI^2^	0.82 ±0.05	1.00 ±0.02	1.07 ±0.07	1.43 ±0.04	1.41 ± 0.04
CSI^3^	0.074 ±0.002	0.092 ±0.001	0.094 ±0.001	0.101±0.002	0.103 ± 0.002
Mortality[%]		0.74 ± 0.18	0.41 ± 0.27	2 ± 0.23	2 ± 0.21
Muscle fat content^4^[%]		4.2^*a*^±0.1	3.9^*b*^±0.1	6.5±0.4	6.8±0.1

Values within the same row with different subscripts are significantly different (*p*≤ 0.05, t-test). Mean ± SEM, the statistical unit is the mean of the net pen (*n* = 3). 19 fish were used to calculate the net pen mean.

^1^Condition factor (CF) = $\frac{1000\times \text{weight}[g]}{\text{length}{\left[g\right]}^{3}}$.

^2^Liver index (LI) = $\frac{\text{liver}-\text{weight}[g]}{\mathrm{weight}[g]}\times$ 100.

^3^Cardio somatic index (CSI) = $\frac{\text{heart}-\text{weight}[g]}{\text{weight}[g]}\times$ 100.

^4^Muscle samples (NQC: Norwegian quality cut) from 10 fish/cage were pooled to analyse the muscle fat content.

### Microarray analysis

**Figure 1:**
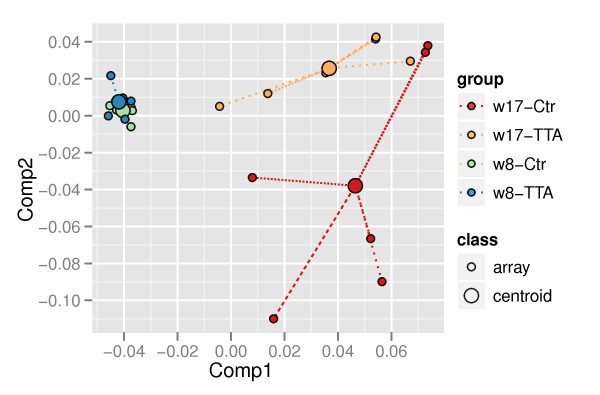
**Correspondence Analysis (CA).** CA of arrays from the two dietary groups for both sampling points (*8.weeks* and *17.weeks*), *n* = 6.

RNA cardiac samples from six individual fish from each dietary group and sampling point were used in the microarray analysis, utilizing the Atlantic salmon SIQ2 microarray [18] in a one-color setup, resulting in a total of 24 arrays. After normalization and filtering, 13166 probes (63%) were classified as present. To obtain a global overview of the general structure of the dataset we applied correspondence analysis (CA) as an explorative technique [19]. The first 2 components of the CA are displayed, together explaining 67% of the total inertia of the different samples (Figure 1). The analysis shows a clear distinction between the two sampling points at *8.weeks* and *17.weeks*. It further shows a relatively dense cluster at the *8.weeks* point with minor separation between samples from the TTA and control group. Even though it was impossible to draw a straight line to separate between the TTA and control samples at the *17.weeks* sampling point, we observed a clear tendency of separation.

To identify differentially expressed (DE) probes in the data set we used moderated *t*-statistics [20], comparing samples from TTA to control fed Atlantic salmon for each sampling point.

### Sampling point: *8.weeks*, end of TTA feeding

**Figure 2:**
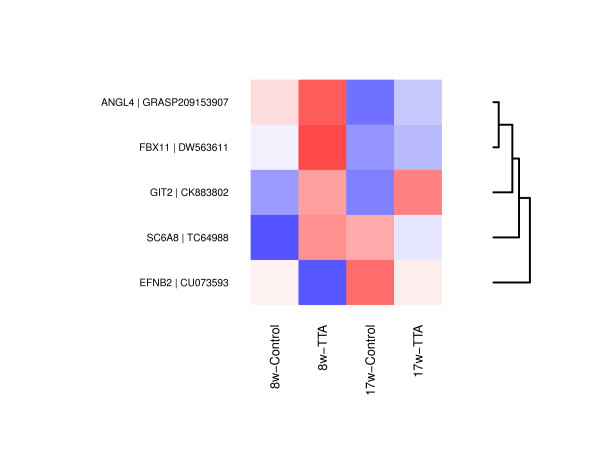
**Heatmaps of genes DE at *8.weeks*.** Columns display the mean log_2_ signal of the biological replicates (*n* = 6), rows display the genes (probe sets) showing DE. Rows were scaled and ordered by hierarchical clustering using euclidean distances (indicated by the dendrogram).

At the *8.weeks* sampling point, five genes were found to be DE between the TTA and the control fed group (Figure 2). The genes were: *Ephrin-b2*, *arf gtpase-activating protein* (*git2*), *f-box only protein 11* (*fbx11*), *angiopoietin-related protein 4* (*ANGPTL4*) and *sodium- and chloride-dependent creatine transporter 1* (*sc6a8*). *Ephrin-b2* was the only gene found to be down-regulated in the TTA fed group.

*Ephrin-b2* (*Efnb2*) in mammals has been reported to be highly expressed in the heart and serves also as a marker for angiogenesis [21]. The protein GIT-2 participates in pleiotropic cellular processes like cell migration and T-cell activation; however, a function affecting the structure of the cytoskeleton [22] may be relevant in our study. Cardiomyocytes rely solely on the creatine transporter sc6a8 for the uptake of creatine from the plasma. Over-expression of the creatinfabe transporter in mice has been reported to correlate with the myocardial creatine content, but also to be associated with cardiac hypertrophy [23]. Angiopoietin-related protein 4 possesses a role in regulating angiogenesis and is also known as a target gene for PPARs and acts as an important stimulator of lipid metabolism [24].

### Sampling point: *17.weeks*, 9 weeks post TTA feeding

1198 probes (930 genes) were found to be DE between the TTA and the control fed group at the *17.weeks* sampling point. In order to facilitate a functional interpretation of the vast number of DE genes, we tested them for enrichment (over-representation) of GO terms from the category “biological process” [25], using conditional hypergeometric testing [26]. To ensure that one gene was represented by a maximum of one probe [26], probes matching the same gene were collapsed prior to hypergeometric testing. This step reduced the total number of probes in the data set from 11143 to 7659 probes and the number of DE probes from 1198 to 930 probes (930 genes). From these 930 genes, 90% showed higher gene expression in the samples from TTA fed fish.

Conditional hypergeometric testing revealed significant over-representation of 36 GO terms. To simplify interpretation, significant GO terms were grouped into five categories according to their function in the heart (Table 2). The grouping was further supported by a strong gene overlap between the different GO terms within the categories (Additional file 2: Figure S1 and Additional file 3: Table S2 ). Overall, the results from the enrichment analysis suggests an increased capacity of heart ventricles from TTA fed Atlantic salmon to catabolize lipids and glycogen. Further, an increased capacity for cardiac contractility and cardiac tissue morphogenesis is indicated.

**Table 2:** **Gene Ontology enrichment analysis for sampling point *17.weeks***

**GOID**	***p*-value**	**Count**^**1**^	**Size**^**2**^	**Gene ontology term**
**Fat metabolism**				
GO:0046395	5.33e-04	24	96	carboxylic acid catabolic process
GO:0034440	5.69e-03	13	49	lipid oxidation
GO:0044242	8.41e-03	19	86	cellular lipid catabolic process
**Heart performance**				
GO:0006936	1.56e-03	37	182	muscle contraction
GO:0008015	5.56e-03	38	202	blood circulation
GO:0003015	6.45e-03	22	102	heart process
GO:0008016	7.18e-03	17	73	regulation of heart contraction
GO:0055008	8.94e-03	12	46	cardiac muscle tissue morphogenesis
**Citrate cycle (TCA)**				
GO:0009109	1.87e-07	15	28	co-enzyme catabolic process
GO:0006084	2.12e-07	18	39	acetyl-CoA metabolic process
GO:0006099	2.32e-07	14	25	tricarboxylic acid cycle
GO:0006091	9.39e-05	46	214	generation of precursor metabolites and energy
GO:0045333	3.49e-04	23	88	cellular respiration
GO:0044248	1.34e-03	36	180	cellular catabolic process
**carbohydrate metabolism**				
GO:0006112	6.21e-04	16	54	energy reserve metabolic process
GO:0016052	1.17e-03	19	73	carbohydrate catabolic process
GO:0006073	1.27e-03	14	47	cellular glucan metabolic process
GO:0046164	1.77e-03	16	59	alcohol catabolic process
GO:0019320	2.97e-03	14	51	hexose catabolic process
GO:0006096	3.26e-03	12	41	glycolysis
**Other**				
GO:0007338	1.27e-03	14	47	single fertilization
GO:0001824	1.59e-03	12	38	blastocyst development
GO:0051246	2.00e-03	86	514	regulation of protein metabolic process
GO:0010171	2.83e-03	20	84	body morphogenesis
GO:0055114	3.60e-03	40	210	oxidation reduction
GO:0007050	3.67e-03	19	80	cell cycle arrest
GO:0043009	5.00e-03	48	267	chordate embryonic development
GO:0001822	5.64e-03	19	83	kidney development
GO:0040010	6.35e-03	33	171	positive regulation of growth rate
GO:0009790	7.54e-03	124	815	embryo development
GO:0001655	8.41e-03	19	86	urogenital system development
GO:0070585	8.44e-03	10	35	protein localization in mitochondrion
GO:0044265	8.45e-03	66	399	cellular macromolecule catabolic process
GO:0007018	9.35e-03	16	69	microtubule-based movement
GO:0006839	9.35e-03	16	69	mitochondrial transport
GO:0006402	9.66e-03	13	52	mRNA catabolic process

^1^Number of times the GO term is represented in the list of DE genes, only GO terms having ≥ 10 genes were considered.

^2^Number of times the GO term is represented in the filtered list of genes on the array.

#### Heart performance

The group fed TTA showed an up-regulation in the expression of genes encoding contractile proteins like myosin heavy chain 6 (MYH6), myosin light chain (MYL9), cardiac myosin binding protein (MYBPC3), cardiac troponin (TNNT2), myomesin-1 (Myom1) and actin (ACTA1, ACTA2).

Moreover, the same group showed increased expression of the cardiac homeodomain factor *Nkx2.5* and the iroquois-related homeobox factors 3 and 5 (*irx3*, *irx5*). Nkx2.5 and the iroquois transcription factors have been reported to control cardiac morphogenesis and growth [27,28]. Furthermore, we observed increased expression of *FK506 binding protein 1A and 1B* (*FKBP1A*, *FKBP1B*) and *N*^*a* + ^/^*K* + ^*-transporting ATPase subunitα3* (*ATP2A2*), encoding an ion-pump responsible for establishing and maintaining the electrochemical gradients at the plasma membrane of the cardiomyocyte. Decreased amounts of this transporter were found in biopsies from humans suffering heart failure [29]. FKBP1A and B are known to interact with intracellular calcium-release channels. In cardiomyocytes FKBP1B is a binding partner for the major Ca^2 + ^release channel ryanodine receptor 2 (RyR2). RyR2 is required for the Ca^2 + ^-induced Ca^2 + ^ release from the sacroplasmatic reticulum (SR) causing activation of the contractile proteins. Binding of FKBP1B to RyR2 results in channel closure. Mice deficient for FKBP1B showed no divergence in the normal cardiac phenotype under normal conditions but showed exercise-induced arrhythmias [30]. In relation to Ca^2 + ^ signaling, we also found the Na^ + ^/Ca^2 + ^exchanger *SLC8A1* to be up-regulated. We also found an increased expression of the SR Ca^2 + ^ ATPase 2 (*ATP2A2* also known as *SERCA2*), encoding an SR calcium pump that is a key component of the cardiac excitation-contraction mechanism [31].

The *Kv channel interacting protein 1* (*Kcnip1*) was found to be down-regulated. The protein Kcnip1 is an integral part of the multimeric Kv4 channel complex, and important for modulating the K-flux across this channel by causing a shortening of the cardiac action potential [32]. Prolongation of the cardiac action potential on the other hand, potentially caused by decreased Kcnip1 expression, is associated to cardiac hypertrophy [33].

In summary, the results suggest an increased cardiac hypertrophy together with increased potential for cardiac contractility, as indicated by the higher transcription of the various ion channels/pumps and contractile proteins.

#### Fat metabolism

**Figure 3:**
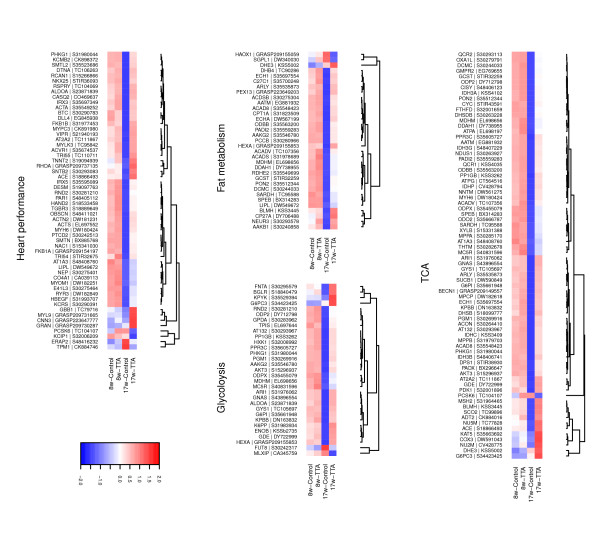
**Heatmaps of genes DE at *17.weeks.*** Unique genes from the different categories (Table 2). Columns display the mean log_2_ signal of the biological replicates (*n* = 6), rows display the genes (probe sets) showing DE. Rows were scaled and ordered by hierarchical clustering using euclidean distances (indicated by the dendrogram).

Nearly all of the genes in this group were up-regulated (Figure 3). We found up-regulation of the mitochondrial trifunctional protein *HADHA*, the mitochondrial fatty acid transporter carnitine palmitoyltransferase (*Cpt1a*), *lipoprotein lipase* (*Lpl*), the mitochondrial acyl-CoA dehydrogenases (*ACADS*, *ACADV * and *ACADSB*) and of *peroxisomal multi-functional enzyme type 2* (*Hsd17b4*). We also observed an up-regulation of *malonyl-CoA decarboxylase* (*MLYCD*). Malonyl-CoA is a potent inhibitor of CPT1 and thus crucial in regulating the transport of fatty acids into the mitochondria for catabolism. Malonyl-CoA decarboxylase has been reported to function as a positive regulator of cardiac fatty acid oxidation by decreasing the levels of the CPT1 inhibitor malonyl-CoA [34]. Thus, the results indicate increased fatty acid oxidation capacity in cardiac ventricles from TTA fed Atlantic salmon.

#### Glycolysis

The genes of the six GO terms that were grouped together contained almost entirely genes encoding enzymes or subunits participating in glycolysis. We found increased expression of *hexokinase 1* (*HXK1*), the phospho-fructokinases *aldolase A* (*ALDOA*) and *6-phosphofructokinase type C* (*K6PP*), *glycerol-3-phosphate dehydrogenase* (*GPDA/GAPDH*) and the pyruvate dehydrogenases *DLAT* and *OPDX*. In accordance, we also observed an increased expression of *MLX-interacting protein* (*MLXIP*), which has been suggested to be an essential regulator of cellular glycolysis [35]. All of the genes showed increased transcription, therefore clearly indicating increased glycolysis in the hearts from TTA fed Atlantic salmon.

#### Tricarboxylic acid (TCA) cycle

As in the previously described categories, almost all of the genes in this category showed an increased expression. The proteins encoded by nearly all of the genes in this category are part of the TCA-cycle. For a graphical representation of the genes within the TCA cycle, see Additional file 4: Figure S2.

### qRT-PCR

**Figure 4:**
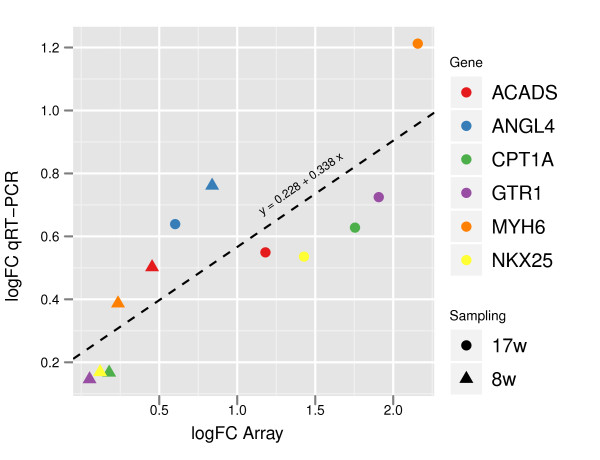
**qRT-PCR verification.** Comparison of the log_2_FC data for the two sampling points between Microarray and qRT-PCR measured samples. qRT-PCR data was normalized to the expression of the housekeeping gene EF1*α*. Pearson correlation; r = 0.8314, p = 0.0008. The dotted line indicates the linear regression line.

To validate the microarray results, six genes were analyzed by qRT-PCR between the TTA and control fed group for the *8.weeks* and *17.weeks* samples, using the same RNA samples that were used in the microarray experiment. The results showed a significant correlation between the logFCs obtained by qRT-PCR and those obtained by microarray (Pearson correlation *r* = 0.8314; *p* = 0.0008; Figure 4).

**Figure 5:**
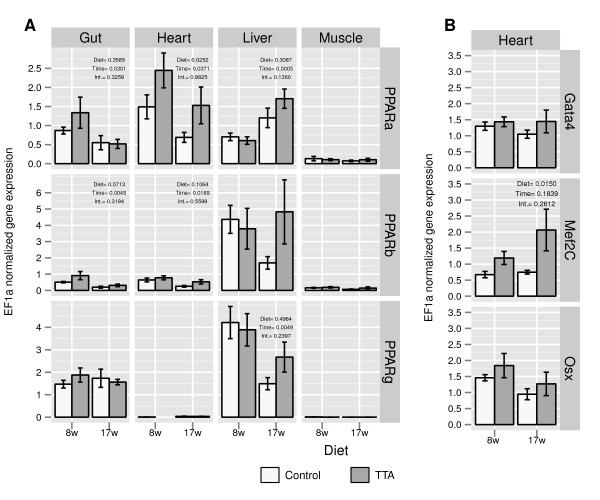
**Gene expression.A**: Expression of PPAR*α*, *β * and *γ * in Atlantic salmon fed TTA or a control diet. The different tissues liver, heart, muscle and gut were sampled at two sampling points (*8.weeks* and *17.weeks*). **B**: Cardiac expression of ***Gata4***, *Myocyte-specific enhancer factor 2C***(Mef2C)** and ***Nkx2.5*** in Atlantic salmon fed TTA or a control diet. Gene expression was normalized to the expression of the housekeeping gene EF1*α*. The *p*-values for the effects of Diet, Time and Interaction from the two way ANOVA are displayed in the upper right corner. Data are presented as means ± SEM, *n* = 6.

In addition, we measured the gene expression of the three PPAR subtypes *α*,*β* and *γ* in control and TTA fed Atlantic salmon in the four different tissues: Heart, muscle, liver and gut (pyloric caeca) from both sampling points (Figure 5A). Analyzing the expression levels using analysis of variance (ANOVA) showed that only the *PPARα*expression in the heart was significantly increased in the TTA group. The gene expression levels of PPAR*γ* in heart and muscle were too low to allow reliable quantification. Since the microarray data revealed quite clearly that TTA affected genes are involved in regulating the heart performance, we used qRT-PCR to measure the expression of the cardiac transcription factors *GATA4*, *Mef2C* and *osteonectin* (*Osx*). For all three transcription factors we observed a trend of higher mean transcription in the TTA group (Figure 5B), however, only *Mef2C* showed statistical significance.

**Figure 6:**
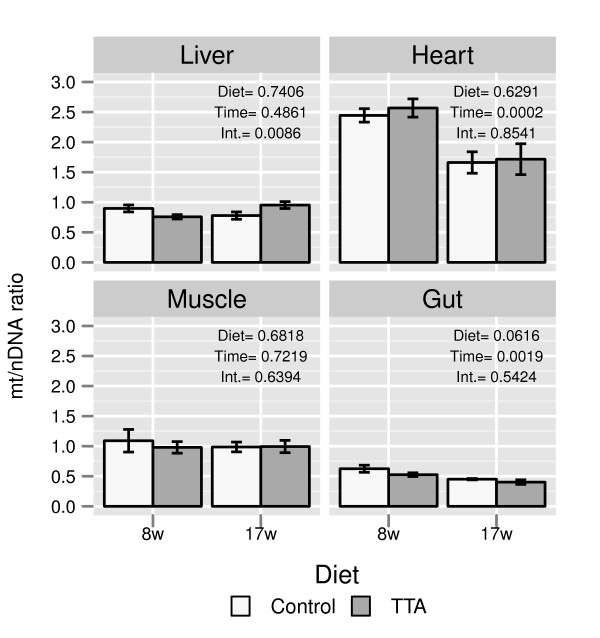
**Mitochondrial biogenesis.** Effect of feeding TTA on the ratio between mitochondrial and nuclear DNA in the different tissues. The p-values for the effects of Diet, Time and Interaction from the two way anova are displayed in the upper right corner. Data are presented as means ± SEM, *n* = 6.

TTA has previously been reported to stimulate mitochondrial biogenesis in mammals [36]. In this study TTA had no effect on mitochondrial biogenesis as measured by the ratio of mt/nDNA (Figure 6). In the liver we found a significant interaction between dietary treatment and time for mitochondrial biogenesis.

## Discussion

In the present study we investigated the response of Atlantic salmon to TTA during the seawater phase. The results from our study show that feeding TTA had profound effects on the cardiac gene expression at sampling point *17.weeks*, 9 weeks after TTA feeding ended. The level of TTA applied in the study was, with 0.25%, lower than previous studies that have been conducted in Atlantic salmon (compare [1,6,37]). The mortality rates previously observed in Atlantic salmon in response to high TTA levels was not observed in this study.

Gene set over-representation of the transcription profile at *17.weeks* shows an increased capacity of fat catabolism, glycolysis and activity of the TCA cycle as well as cardiac contractility and cardiac hypertrophy. Overall, the results suggest a scenario where cardiac ventricles of TTA pre-fed fish are able to generate more energy via a TCA-cycle that is fueled by metabolites from fat catabolism and glycolysis. TTA functions as a ligand for all three PPAR subtypes [3,4], which have crucial functions in the transcriptional regulation of cardiac metabolism. In mice the transcriptional effects of TTA in the heart have been shown to be mediated almost exclusively via PPAR*α*[38]. Gain-of-function and loss-of-function mutations have shown that PPAR*α* is a crucial transcription factor in the cardiac metabolism, regulating mainly cardiac fatty acid uptake and oxidation [10,11]. Furthermore, activation of PPAR*α*has been demonstrated to shift cardiac energy utilization away from glucose and towards fatty acid oxidation, actually mimicking the cardiac phenotype observed in diabetic hearts [11]. Interestingly the cardiac phenotype of PPAR*β* differs from that of PPAR*α*, indicating that both transcription factors regulate, at least partly, different subsets of genes in the heart. PPAR*β* loss-of-function hearts suffer from myocardial lipid accumulation and cardiomyopathy [13]. Gain-of-function mutations on the other hand clearly show that PPAR*β* positively regulates cardiac glucose utilization [12], and also stimulates cardiac growth [39]. Thus, the significantly higher cardiac transcription of PPAR*α* and the elevated mean transcription of PPAR*β*/ in concert with the activation of their down-stream pathways, fat catabolism and the glycolysis pathway suggest that cardiac effects of TTA in Atlantic salmon are mediated by both PPAR*α* and PPAR*β*. Intriguingly, over-expression of a constitutively active form of PPAR*β*in murine skeletal muscle has been reported to mimic training-based muscle adaptation [40]. Hence, it has been speculated, in accordance with the results from PPAR*β* over-expression in mice [12], that PPAR*β*causes “physiological” cardiac hypertrophy [39].

Between the *8.weeks* and *17.weeks* sampling points, the hearts grew by a considerable portion in absolute and relative terms. The gene expression profile in hearts of TTA fed fish at *17.weeks* suggests that the cardiac growth of TTA fed fish is shifted towards “physiological” hypertrophy, which may translate to an increased cardiac output. This notion is supported by the expression profile found for the category “cardiac performance” at *17.weeks*, unanimously pointing to an increased cardiac contractility and also showing up-regulation of crucial cardiac transcription factors. In particular the higher transcription of the cardiac transcription factors Nkx2.5 and Mef2C can be regarded as markers for cardiac hypertrophy/growth. It has been demonstrated in mice that over-expression of Mef2C is sufficient to induce cardiac hypertrophy [41]. Furthermore, both Mef2C and Nkx2.5 have been shown, *in vitro*, to be regulated by PPAR*α* in cardiomyocytes [42].

It should also be noted that although we did not find significant differences in relative heart weight in this study, in other studies we found that TTA significantly increases heart size in Atlantic salmon [8,9], and that the effect seems to be correlated to the dose of TTA (Rørvik, unpublished data). Thus, it is tempting to speculate that the increase in relative heart weight may be related to the cardiac transcriptional changes induced by TTA. A “cardiac exercise” stimulating effect is of high relevance for salmonid aquaculture. Atlantic salmon, having a circulatory system that is naturally adapted to long migration routes and high activity, show alteration in cardiac morphology and a reduced relative heart weight in captivity [43]. In addition, circulatory failure has been identified as an important cause of mortality in salmon farming [44]. Thus, using TTA may be one way to support the cardiac performance of fish in captivity.

The highest tissue concentrations of TTA in Atlantic salmon, as well as in mice, can be found in the heart [1,45]. In accordance, the heart was also the tissue where the strongest transcriptional response of PPAR*α*was detected. The main transcriptional effects were found nine weeks after the TTA feeding stopped and where our data suggested that the cardiac tissue levels of TTA were neglectable. However, we have no information about the course of gene expression between both sampling points, thus it might very well be that the effects of sampling at *17.weeks* are the remains of earlier, stronger transcriptional effects. It is remarkable that a similar, delayed response in expression of lipid metabolism related genes to TTA has been observed in our previous Atlantic salmon studies [8,9], indicating a common underlying mechanism. It is possible that the delay in transcriptional response is caused by a common, yet unknown, mechanism.

## Conclusions

In conclusion, based on results from microarray analysis, this study demonstrates that TTA increases cardiac fatty acid oxidation and glycolysis as well as contractility and cardiac hypertrophy in Atlantic salmon. The gene expression profiles further favor a scenario of “physiological” hypertrophy in response to TTA. This increased cardiac efficiency may offer significant benefits in situations with increased oxygen demand.

## Methods

### Feeding trial

The experiment was conducted at Nofima Marin sea-water research station, Averøy, western Norway. Atlantic salmon used in this experiment were hatched at Nofima Marin research station (Sunndaløra, Norway) one year earlier (S1/1+ Salmon). The experiment started with the seawater transfer of the fish on the 15th of May 2007 and lasted until the 27th of September 2007. A randomized block design with triplicate seawater net-pens and 400 fish per pen (pen = 125 m^3^) was used for the experiment. Control and TTA diets (0.25% (w/w) TTA (Thia Medica, Norway)) were produced by Biomar (Biomar AS, Myre, Norway). Both TTA and control diets were fed to the fish until the 16th of July 2007, from this point until the end of the experiment only the control diet was fed to the fish. Low levels of TTA (0.25%) and a short feeding period were chosen in order to avoid negative TTA effects (mortality, altered kidney morphology [1,46]). TTA was fed for the first eight weeks after sea transfer, a period where the physiology of the salmon alters due to changing from a fresh to a saltwater environment; and we speculated that an increased capacity for energy utilization may be beneficial. Fish were sampled from the cages to represent the average fish weight for the cage. Sampling was done on the following dates: 16-18th of July 2007 (sampling point: *8.weeks*, end of the TTA feeding period) and 25-27th of September 2007 (sampling point: *17.weeks*). For each sampling point, fish were sampled for: heart ventricle, liver, muscle and gut (pyloric caeca). The tissue samples were snap frozen in liquid nitrogen and stored at -80°C.

### Fat analysis

Fat content in the muscle (Norwegian quality cut–NQC, Norwegian standard procedure - NS 9401 1994) was measured in pooled samples (10 fish) from each net pen as described in [47]. TTA was measured within the total cardiac lipids. For the analysis 10 ventricles from Atlantic salmon out of the same net pen were pooled. Total heart lipids were extracted with chloroform-methanol [48] and fatty acid methyl esters (FAME) were obtained by heating of lipids with methanol at 90°C/1 hour, where *H*_2_*SO*_4_ was used as a catalyst [49]. After extraction into an organic solvent, the FAME were analyzed by gas-liquid chromatography. A gas chromatograph GC 8000 TOP (Finnigan, USA) was equipped with a programmed temperature vaporization (PTV) injector, flame-ionization detector (FID), AS 800 autosampler and a fused silica capillary column coated with dimethylpolysiloxane stationary phase, DB1-ms (J & W Scientific, USA). Hydrogen was used as a carrier gas. Column temperature was programmed from 110 to 310°C with a gradient 2.5°C/min. The GC signal was acquired using Chromeleon software (Dionex, USA). Peaks were identified by means of known FA standards (Larodan Fine Chemicals, Sweden and Sigma-Aldrich, USA) and by means of mass spectra, obtained by GC/MS analysis (GCQ, Finnigan, USA) on the same column. An internal standard (C21:0) was used for quantitation after calibration with known mixtures of FA standards.

### RNA extraction

Two individual samples from each one of the 3 net-pens were samples for heart ventricle, liver, muscle and gut. The samples were randomly chosen (*n*=6 per dietary group and sampling point) and homogenized using a rotor tissue lyser (Precellys 24, Bertin technologies, France). Total RNA was extracted and purified using column purification (96 universal Tissue Kit, Qiagen, Hilden, Germany) according to the manufacturer’s instructions. Traces of genomic DNA in the samples were eliminated by on-column-DNase digestion (Qiagen). RNA concentrations were measured for all samples using a NanoDrop 1000 Spectrophotometer (Thermo Fisher Scientific, Wilmington, USA). RNA quality for samples later used in the microarray was determined using a Agilent 2100 Bioanalyzer (RNA 6000 NanoLabChip, Agilent, Waldborn, Germany).

### Microarray hybridization

A customized oligo (60-mer) Atlantic salmon microarray in the 4x44K format (Agilent, [18]) was used to detect differential gene expression between samples from the heart ventricles of control and 0.25% TTA fed fish for the *8.weeks* and *17.weeks* sampling points. The array contained 21012 different probes spotted in duplicates. RNA samples from individual fish were hybridized to the microarray. 24 individual microarrays were performed using 12 fish (6 control and 6 TTA fish) at the 8 week sampling point, and similarly at the 17 week sampling point. All RNA samples used in the hybridization had RIN values ranging from 9.5 to 10. 500ng RNA were amplified and labeled with Cy3 using the Quick Amp Labeling Kit (One Color-Agilent). After purification the cRNA was quantified using NanoDrop. Subsequently 1.65*μ*g Cy-dye-labeled cRNA was fragmented (mean size, approximately 50-100 nucleotides) with fragmentation buffer (Agilent Technologies) at 60°C for 30 min; cRNA was subsequently hybridized to the microarray at 65°C for 17 h. All steps were conducted according to the Agilent protocol (One-Color Quick Amp Labeling, Version 5.7). The microarray chips were scanned using a Agilent Microarray Scanner (G2565CA) and analysis of the microarray images was done in Agilent’s Feature Extraction Software (Version 10.5.1.1) using the one-color (GE1_105_Dec08) protocol.

### Microarray analysis

Normalization and analysis of the data was performed in R/Bioconductor [50,51] using the “limma” package [20]. The background corrected fluorescence signals (gProcessedSignal) were obtained from Feature Extraction (Agilent). Spots were filtered according to the following criteria provided by Feature Extraction: gIsFound, gIsPosAndSignif and gIsWellAboveBG (a description of the parameters can be found in the Feature Extraction Software Reference Guide). The mean signal of the duplicated probes was calculated and all control spots together with probe sets showing more than three missing values were removed from the dataset. The data was subsequently normalized using quantile normalization in order to adjust the scale of intensities across arrays [52]. After normalization the signals were log_2_ transformed. The normalized/filtered dataset then contained 13166 probe sets (63% of the total). The raw and normalized data is publicly available at NCBI’s GEO repository ( http://www.ncbi.nlm.nih.gov/geo/, AccNr: **GSE25305**). Differential expression of probe sets was assessed by fitting a linear model, including the effects of feeding (2 levels: Control and TTA) and the effects of sampling point (2 levels: *8.weeks* and *17.weeks*) and their interaction. The specific comparisons: TTA *vs.* Control at sampling point *8.weeks* and TTA *vs.*Control at sampling point *17.weeks* were made by extracting the appropriate contrasts from the linear model. For each contrast moderated *t*-statistics were calculated using an empirical Bayes method [53]. Probes without annotation were removed from the dataset before controlling the false discovery rate [54] simultaneously across probe sets and contrasts (method: “ global” in the limma function “decideTests”). Probe sets with a *q*-value ≤ 0.05 and a log_2_FC ≥ 0.5 were declared DE for the corresponding contrast. A comprehensive list of all DE probes for each contrast can be found in Additional file 5: Table S3.

Probe annotation and GOs were retrieved using the top Blast function implemented in Blast2GO [55]. Full length probe sequences were blasted against protein sequences from the Swissprot database in a BlastX search. The E-value cut off was set to 10^−6^. Hypergeometric testing for over-representation of GO terms from the category biological process [25] among the genes DE for the contrast TTA *vs.*Control at the *17.weeks* sampling point was conducted using the GOstats package [26]. Before testing, probes matching to the same gene were collapsed to the probe showing the largest variance.

Correspondence analysis was conducted using the R package “made4” [56]. Probe sets with missing values were removed from the dataset prior to correspondence analysis.

### Quantitative RT-PCR

Single strand cDNA was synthesized from 500ng of total RNA using oligo dT primers and the Taq Man reverse transcription Kit (Applied Biosystems, CA, USA). qRT-PCR was performed on a Light-Cycler 480 (Roche, Switzerland). For the PCR reaction, 2x SYBR green I master Mix (Roche), 0.41nM of each primer and the cDNA template were mixed in a total reaction volume of 10*μ*l. Primer sequences are listed in Table 3. A three step PCR protocol with 45 cycles (15s 95°C, 15s 60°C, 15s 72°C) was used. To verify specific amplification, a melting curve analysis step was done at the end of the program. In order to verify the results obtained through the microarray experiment, the same 24 samples used in the array were used in a qRT-PCR approach. Six genes were then randomly picked and samples were analyzed in duplicates. The expression level was calculated using the standard curve method (Applied Biosystems User Bulletin 2). The standard curve was produced from a serial dilution of a pool consisting of all cDNA samples. The expression levels were standardized to the expression of the housekeeping gene *elongation factor 1α*(*EF1α*, [57]).

**Table 3:** qRT-PCR primer sequences

**Gene**	**Accession no.**	**Forward primer (3’ - 5’)**	**Reverse primer (5’ - 3’)**	
mt D Loop B (gDNA)	NC001960	CCCCTGAAAGCCGAATGTAA	CGACCTTGTTAGACTTCTTTGCTTG	
MyoD2 (gDNA)	AJ557150	CAGAGCCAGGATTACACTCGTTACA	GCATGTCGCTGGTGTTGAAG	
PPAR*α*	DQ294237	TCCTGGTGGCCTACGGATC	CGTTGAATTTCATGGCGAACT	
PPAR*β*	AJ416953	GAGACGGTCAGGGAGCTCAC	CCAGCAACCCGTCCTTGTT	
PPAR*γ*	AJ416951	CATTGTCAGCCTGTCCAGAC	TTGCAGCCCTCACAGACATG	
EF1*α*	AF321836	CACCACCGGCCATCTGATCTACAA	TCAGCAGCCTCCTTCTCGAACTTC	
MYH6	DW559270	CAGGTCCTCTATGTGCTGGTGTG	TCCTCATTGTAGTTGCTGTCCTCAC	
ANGL4	GRASP209147493	CCGTATGGGGGATGATGCTAA	GGTAGTATGCTGACGACTGACACCT	
GTR1	S30269700	GCCATGGATGTCCTACGTGA	CTCCGCTACATACGGGAAGG	
CPT1A	S31823509	TCCCACATCATCCCCTTCAACT	TGTCCCTGAAGTGAGCCAGCT	
ACADS	S31978689	CTGGGGAAGAAGGAGGACAAG	TCTAGAGCAGCCTGAGCAATACC	
NKX2.5	DW550500	CCCAGTACGTCCACACCCTT	GGAGGTCGGTAAGGCACAGT	
Mef2C	GU252207	CACCGTAACTCGCCTGGTCT	GCTTGCGGTTGCTGTTCATA	
GATA4	HM475152	TCTCCATTCGACAGCTCCGT	CATCGCTCCACAGTTCACACA	
Osx	FJ195614	ATTACTGAGGAGGAGCCCATCATT	CCTCATCCACCTCACACACCTT	

### mt/nDNA ratio

Genomic DNA was isolated from tissue samples from the same individuals as the ones used for the total RNA extraction (totally 96 samples, *n*=6). DNA was isolated using DNAeasy kit (Qiagen) according to the manual. The DNA quality for all samples was checked on a 1% agarose gel and concentration was measured using a NanoDrop Spectrophotometer. The MyoD gene (intron-exon spanning primers) and the mitochondrial D-loop were amplified by qRT-PCR. For the PCR reaction 1x SYBR green I master Mix (Roche), 0.41nM of each primer and the 6.4ng DNA template were mixed in a reaction volume of 10*μ*l. PCR amplification was conducted as described above. All reactions were run in duplicates. Absolute concentrations for mt- and nDNA samples were obtained using the standard curve method. The ratio was calculated by dividing the absolute mtDNA by the absolute nDNA concentration.

### Statistical analysis

All data are presented as means ± SEM with an *n* value as stated. The effect of dietary treatment on the production parameters and qRT-PCR were analyzed by 2-way analysis of variance (ANOVA), using dietary treatment and sampling point as fixed factors and block as a random factor. TTA effects on gene expression and mt/nDNA ratio were calculated using *EF1α*standardized expression values in a 2-way ANOVA with dietary treatment and time as fixed factors. Unless otherwise stated the statistical unit is the individual fish. All analyses were conducted using R [50], plots were produced using the R package ggplot [58] and the heatmaps were produced with the R package lattice/latticeExtra [59].

## Competing interests

The authors declare that they have no competing interests.

## Authors contributions

HT,KR and MT conceived the study and designed the experiment. RB provided the TTA measurements. FG conducted the lab experiment, data analysis and drafted the manuscript. All authors read and approved the final manuscript.

## Supplementary Material

Additional file 1 Table S1. TTA measurements in the cardiac ventricles. Click here for file

Additional file 2 Figure S1. Overlap matrix of the genes from the 36 GO terms that were significantly over represented in TTA fed Atlantic salmon at sampling point *17.weeks*. Rows and columns are hierarchical clustered (indicated by the dendrogram) based on euclidean distance. Overlap is indicated by red color. Click here for file

Additional file 3 Table S2. Full list of DE genes associated to over-represented GO Biological processes terms at sampling point *17.weeks*. Click here for file

Additional file 4 Figure S2. KEGG Pathway diagram. Seven genes from the category TCA (Table 2) could be annotated to a KEGG Ontology (KO) using the program KAAS [60]. These 7 genes were highlighted (yellow/red) in the KEGG reference pathway: TCA-cycle (ko:00020). The genes were annotated to the following enzymes: Citrate synthase [EC:2.3.3.1] - 1 gene; Isocitrate dehydrogenase [EC:1.1.1.42] - 1 gene; Isocitrate dehydrogenase (NAD+) [EC:1.1.1.41] - 3 genes; Succinate dehydrogenase (ubiquinone) flavoprotein subunit [EC:1.3.5.1] - 1gene; and membrane anchor unit [EC:1.3.5.1] -1 gene. Click here for file

Additional file 5 Table S3. Full list of the DE genes after collapsing. **Column 1**: Probe ID; **Column 2**: log_2_FCs of the contrast TTA vs. Control for sampling point *8.weeks*; **Column 3**: log_2_FCs of the contrast TTA vs. Control for sampling point *17.weeks*; **Column 4-6**: Gene annotation: Gene name, gene symbol and e-value. **Column 7-8**: Significance of the corresponding gene for the corresponding contrast. **Column 9**: Joint between GeneID and Symbol, as used in the heatmaps. Click here for file
