# Supplementary material for: Genome wide response to dietary tetradecylthioacetic acid supplementation in the heart of Atlantic Salmon (Salmo salar L)
Source: BMC Genomics. 2012 May 11;13:180. doi: 10.1186/1471-2164-13-180 (PMC3483216; doi:10.1186/1471-2164-13-180)
Supplement: Additional file 1 — Table S1. TTA measurements in the cardiac ventricles. [file 1471-2164-13-180-S1.pdf]

	Start	8.weeks			17.weeks	
		Contr	0.25%TTA	0.5%TTA	Contr	0.5%TTA
C10:0	2.1 ± 0.3	1.6 ± 0.2	1.1 ± 0.3	2.4 ± 1.6	1 ± 0.1	1.2 ± 0.1
C12:0	4.1 ± 0.3	3.6 ± 0.1	3.2 ± 0.2	4.9 ± 0.7	4 ± 0.7	4.2 ± 0.4
C14:1n-5	1.9 ± 0.1	2.7 ± 0.1	2.3 ± 0.1	3.4 ± 0.6	2.3 ± 0.4	2.3 ± 0.2
C14:0	422.6 ± 20.1	487.2 ± 15.3	404.1 ± 14.5	498 ± 58.8	516.2 ± 45.1	475.5 ± 14.8
C15:0i	11.5 ± 0.7	13.3 ± 0.5	11.3 ± 0.4	13.8 ± 1.7	10.8 ± 1.2	10.5 ± 0.5
C15:0ai	3.9 ± 0.3	4.6 ± 0.1	3.9 ± 0.2	4.7 ± 0.5	4 ± 0.4	3.8 ± 0.1
C15:0	50.8 ± 1.5	48.7 ± 1.1	42 ± 1.7	47.3 ± 3.9	48.7 ± 1.8	44.7 ± 0.4
C16:0i	5.4 ± 0.3	5 ± 0.1	4.3 ± 0.1	5.6 ± 0.6	5 ± 0.5	4.8 ± 0.1
C16:1n-9	33.1 ± 1.2	27.1 ± 0.6	24.7 ± 0.9	28.4 ± 2.6	23.2 ± 1	20.7 ± 1
C16:1n-7	469.2 ± 18.6	576.4 ± 15.7	475.4 ± 10.7	567.5 ± 54.2	639.4 ± 54.2	586.3 ± 13.8
C16:1n-7t	2.6 ± 0.1	2.6 ± 0.1	2.2 ± 0.1	2.8 ± 0.4	3.2 ± 0.6	3.7 ± 0.1
C16:1	25.1 ± 0.9	27.8 ± 0.5	25.1 ± 0.7	25.9 ± 2.1	24.5 ± 1.2	22.8 ± 0
C16:0	4397.7 ± 47.7	4371.4 ± 111	4034.4 ± 146.3	3997.4 ± 228.8	4089.6 ± 143.7	3909.4 ± 2.4
C17:0i	19.9 ± 0.8	19.1 ± 0.7	16.4 ± 0.7	17.3 ± 1	17.9 ± 0.9	16.6 ± 0.3
C17:0ai+C17:1n-8	38.6 ± 1.3	39.6 ± 0.8	34.7 ± 1	38.7 ± 2.7	31.9^{a} ± 1.7	29.1^{b} ± 0.2
C17:0	57.3 ± 0.7	55.9 ± 1.5	50 ± 1.3	52.4 ± 2.8	61.4 ± 0.4	57 ± 0.2
C18:3n-6	7.7 ± 1	9.2 ± 0.4	7.6 ± 0.1	9.9 ± 0.9	8.7 ± 0.8	8.3 ± 0.4
C18:4n-3	94.9 ± 11.8	138.7 ± 3	115.2 ± 3.3	149.2 ± 14.3	116.8 ± 10.3	108.7 ± 1.5
C18:2n-6	425.4 ± 20.5	596.9 ± 17.8	511 ± 6.9	552.2 ± 30.4	494.3 ± 29.9	460.9 ± 6
C18:3n-3	154.9 ± 12.1	139.4 ± 7	121.1 ± 4.5	143.6 ± 7.8	100.2 ± 4.3	90.3 ± 1.7
C18:1n-9	2017 ± 60.4	2124.2 ± 65	1888.7 ± 39.8	2088.2 ± 144.9	1745.2 ± 19.1	1560.4 ± 34.9
C18:1n-7	644.6 ± 6.2	672.1 ± 18.9	596.9 ± 13.3	620.4 ± 30.6	666.9 ± 13.6	617.4 ± 4.2
C18:1t	10.3 ± 0.3	9.4 ± 0.2	8.6 ± 0.2	9 ± 0.8	8.3 ± 0.7	8.5 ± 0.1
C18:1	48 ± 0.9	66.4 ± 1.7	66.2 ± 1.7	59.4 ± 3.9	41.4 ± 0.5	37.5 ± 0.8
C18:0	1062.3 ± 12	1036.6 ± 30.6	967.3 ± 29.8	939.5 ± 28.3	1085.6 ± 22.6	1006 ± 5.5
TTA:1n-8	0 ± 0	0^{a} ± 0	1.7^{ab} ± 0.1	4.5^{b} ± 0.7	0 ± 0	0.1 ± 0.1
TTA	0 ± 0	0^{a} ± 0	120.2^{b} ± 1.1	377.8^{c} ± 48.1	0 ± 0	16.5 ± 3.3
C20:4n-6	362 ± 30.8	472.2 ± 22.9	475.9 ± 9.6	505.3 ± 11.9	605.3 ± 23.2	553.9 ± 0.7
C20:5n-3	1409.3 ± 177.9	2230.2 ± 133.2	2183.5 ± 46.2	2339.6 ± 97.8	2777.9 ± 104.5	2646.2 ± 8.2
C20:3n-9	2.7 ± 0.1	2.6 ± 0.2	2.4 ± 0.1	2.5 ± 0.1	2.4 ± 0	2.4 ± 0
C20:3n-6	41.9 ± 3.1	50.4 ± 2	46.7 ± 0.8	45.4 ± 1.2	46 ± 1.8	43.3 ± 0.1
C20:4n-3	150.9 ± 16.7	201.1 ± 6.3	185.3 ± 3.4	184.8 ± 7.5	167.9 ± 10	162.8 ± 1.7
C20:2n-6	49.8 ± 2.3	56.1 ± 0.4	51.8 ± 0.4	51.9 ± 2.2	44.9 ± 0.9	40.9 ± 0.7
C20:1n-11	73.6 ± 3.6	85.5 ± 1.8	82.4 ± 1.2	85.9 ± 4.9	49.8 ± 2.3	47.2 ± 1.6
C20:1n-9	502.5 ± 20.3	1151.5^{a} ± 44.9	1079.6^{ab} ± 17.6	946.2^{b} ± 59	593.7 ± 27.2	563.8 ± 16.8
C20:1n-7	33.1 ± 1	76.2 ± 2.8	70.6 ± 1.3	61.9 ± 3.1	60.3 ± 1.1	56.1 ± 1.7
TTA-SO	0 ± 0	0^{a} ± 0	29.9^{ab} ± 6.3	62.7^{b} ± 11.7	0 ± 0	0.7 ± 0.7
C20:0	25.1 ± 0.5	20.2 ± 0.5	19 ± 0.3	19.2 ± 1	19.2 ± 0.5	17.8 ± 0.5
C21:5n-3	36.1 ± 5.7	52.5 ± 2.6	46.1 ± 1.8	55.6 ± 2.9	64.7 ± 4	65.1 ± 0.4
C22:5n-6	98.2 ± 9.5	129 ± 5.6	124.8 ± 1.9	127 ± 1.7	135.4 ± 6.9	143.6 ± 0.4
C22:6n-3	6745 ± 1037.9	8759.3 ± 719.4	9395.3 ± 310.7	9481 ± 480.3	7946.9 ± 236.3	8011.2 ± 69
C22:4n-6	22.4 ± 2.8	33 ± 2.2	31.5 ± 0.7	36.8 ± 1.3	50.8 ± 3.9	55.5 ± 1.3
C22:5n-3	408.8 ± 57.1	643.9 ± 44.5	630.1 ± 19.2	705.8 ± 29.6	886.7 ± 35.3	867.8 ± 2.5
C22:2n-6	6.9 ± 0.7	7.9 ± 0.2	6.1 ± 0.1	6.2 ± 0.2	6.5 ± 0.2	6.3 ± 0.3
C22:1n-11	413.5 ± 25.7	856.8 ± 49.1	774.3 ± 13	732.7 ± 56.4	484 ± 33.1	453.1 ± 34.9
C22:1n-9	59.4 ± 2.5	141^{a} ± 6.5	127.5^{a} ± 2	111.7^{b} ± 5.1	76 ± 3.6	69.9 ± 5.5
C22:1n-7	9.8 ± 0.2	17.5 ± 1.1	16.8 ± 0.4	15.7 ± 1.2	14.5 ± 0.6	13.7 ± 1
C22:0	7.5 ± 0.3	7.1 ± 0.3	6.3 ± 0.1	6.9 ± 0.4	6.9 ± 0.2	6.4 ± 0.3
C23:0	3.1 ± 0.2	2.8 ± 0.1	2.7 ± 0.2	3.2 ± 0.1	4 ± 0.2	3.8 ± 0.1
C24:1n-9	356.8 ± 4.7	313.4 ± 14.4	314.6 ± 4	304.6 ± 3.3	325.5 ± 13.2	298 ± 12.7
C24:0	7 ± 0.2	6.7 ± 0.3	6.2 ± 0.2	6.3 ± 0.2	7.4 ± 0.3	6.6 ± 0.3
Total FA	20836.1 ± 1548.6	25796 ± 797	25249.1 ± 488.2	26163.3 ± 1108.1	24127 ± 480.6	23243.8 ± 161.9
SFA	6118.8 ± 78.4	6123.3 ± 152.2	5607.1 ± 195.3	5657.6 ± 328.7	5913.6 ± 172.2	5597.5 ± 11.5
SFA w. TTA & TTA-SO	6118.8 ± 78.4	6123.3 ± 152.2	5757.2 ± 195.2	6098.2 ± 375.6	5913.6 ± 172.2	5614.8 ± 7.4
MUFA	4687.7 ± 145.2	6138.5 ± 98.8	5545 ± 54	5651.9 ± 353.5	4746.7 ± 137.6	4349.4 ± 101.1
MUFA with TTA:1n-8	4687.7 ± 145.2	6138.5 ± 98.8	5546.7 ± 53.9	5656.5 ± 354.1	4746.7 ± 137.6	4349.5 ± 101.3
PUFA n-3	8999.8 ± 1319	12165.2 ± 911.9	12676.6 ± 381.4	13059.7 ± 610.9	12061 ± 302.8	11952.1 ± 74.4
PUFA n-6	1014.2 ± 69.9	1354.6 ± 40.8	1255.4 ± 18.4	1334.7 ± 40.6	1391.8 ± 33.1	1312.8 ± 6.4
PUFA n-9	2.7 ± 0.1	2.6 ± 0.2	2.4 ± 0.1	2.5 ± 0.1	2.4 ± 0	2.4 ± 0
Trans-FA	12.9 ± 0.4	12 ± 0.3	10.8 ± 0.3	11.8 ± 1.2	11.5 ± 1.3	12.2 ± 0
PUFA n-3 / PUFA n-6	8.8 ± 0.7	9 ± 0.5	10.1 ± 0.2	9.8 ± 0.5	8.7 ± 0.1	9.1 ± 0.1
Anti-inflammatory index	2353.1 ± 144.3	2468.7 ± 93.8	2575 ± 54.3	2485.2 ± 65.7	1930 ± 69.6	2088.6 ± 11.7
D5 desaturase index (n-3)	9.3 ± 0.2	11.1^{a} ± 0.4	11.8^{ab} ± 0.1	12.7^{b} ± 0.3	16.6 ± 0.4	16.3 ± 0.2
D5 desaturase index (n-6)	8.6 ± 0.2	9.4^{a} ± 0.2	10.2^{b} ± 0.1	11.1^{c} ± 0.2	13.2 ± 0.8	12.8 ± 0
D6 desaturase index (n-3)	0.6 ± 0	1 ± 0	1 ± 0	1 ± 0.1	1.2 ± 0.1	1.2 ± 0
D6 desaturase index (n-6)	0 ± 0	0 ± 0	0 ± 0	0 ± 0	0 ± 0	0 ± 0
D9 desaturase C16:0	0.1 ± 0	0.1 ± 0	0.1 ± 0	0.1 ± 0	0.2 ± 0	0.2 ± 0
D9 desaturase C18:0	1.9 ± 0	2 ± 0	2 ± 0	2.2 ± 0.1	1.6 ± 0.1	1.6 ± 0
C20:5n-3/C18:3n-3	9 ± 0.4	16 ± 0.9	18.1 ± 0.3	16.4 ± 1.2	27.7 ± 0.2	29.3 ± 0.6
C20:4n-6/C18:2n-6	0.8 ± 0	0.8 ± 0	0.9 ± 0	0.9 ± 0.1	1.2 ± 0.1	1.2 ± 0
Elong C18:0/C16:0	0.2 ± 0	0.2 ± 0	0.2 ± 0	0.2 ± 0	0.3 ± 0	0.3 ± 0
Elong n-3 C20:4/C18:4	1.6 ± 0	1.4^{ab} ± 0	1.6^{a} ± 0	1.3^{b} ± 0.1	1.4 ± 0.1	1.5 ± 0
Elong n-6 C20:3/C18:3	5.5 ± 0.3	5.5^{ab} ± 0.2	6.2^{a} ± 0.1	4.7^{b} ± 0.4	5.4 ± 0.3	5.2 ± 0.2
Elong n-7 C18:1/C16:1	1.4 ± 0	1.2^{ab} ± 0	1.3^{a} ± 0	1.1^{b} ± 0.1	1.1 ± 0.1	1.1 ± 0
DBI	2.8 ± 0.2	3.1 ± 0.1	3.2 ± 0	3.2 ± 0.1	3.2 ± 0	3.3 ± 0
DBI with TTA:1n-8	2.8 ± 0.2	3.1 ± 0.1	3.2 ± 0	3.2 ± 0.1	3.2 ± 0	3.3 ± 0
