# Supplementary figures and images for: Genome wide response to dietary tetradecylthioacetic acid supplementation in the heart of Atlantic Salmon (Salmo salar L)
Source: BMC Genomics. 2012 May 11;13:180. doi: 10.1186/1471-2164-13-180 (PMC3483216; doi:10.1186/1471-2164-13-180)

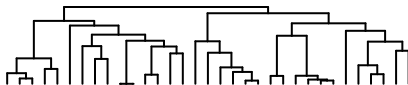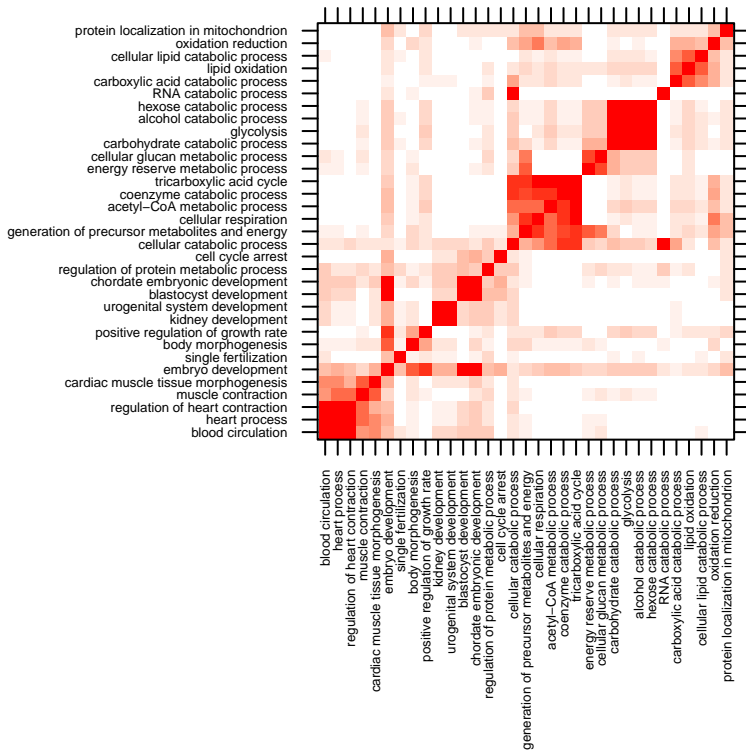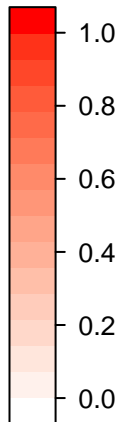

Supplement: Additional file 2 — Figure S1. Overlap matrix of the genes from the 36 GO terms that were significantly over represented in TTA fed Atlantic salmon at sampling point 17.weeks. Rows and columns are hierarchical clustered (indicated by the dendrogram) based on euclidean distance. Overlap is indicated by red color. [file 1471-2164-13-180-S2.pdf]

# CITRATE CYCLE (TCA CYCLE)

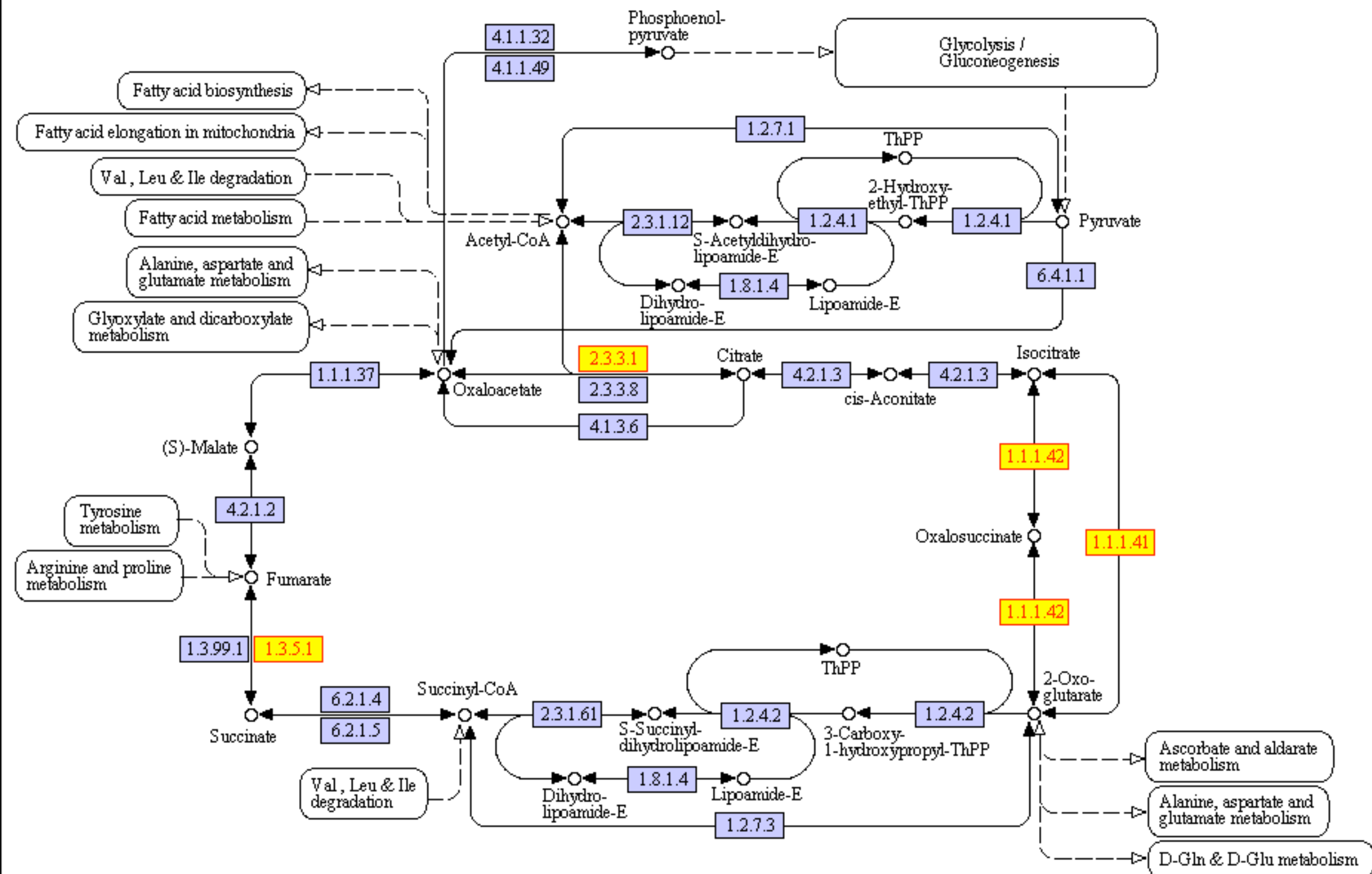

Supplement: Additional file 3 — Table S2. Full list of DE genes associated to over-represented GO Biological processes terms at sampling point 17.weeks. [file 1471-2164-13-180-S3.pdf]
